# Effectiveness of Symptomatic Physiotherapy in Enhancing the Psychological Parameters of a Patient With Guillain-Barré Syndrome: A Case Report

**DOI:** 10.7759/cureus.55389

**Published:** 2024-03-02

**Authors:** Gauri Kariya, Vikrant G Salphale, Ragini Dadgal

**Affiliations:** 1 Community Health Physiotherapy, Ravi Nair Physiotherapy College, Datta Meghe Institute of Higher Education and Research, Wardha, IND; 2 Neuro-Physiotherapy, Mahatma Gandhi Mission (MGM) School of Physiotherapy, Wardha, IND; 3 Neuro-Physiotherapy, Ravi Nair Physiotherapy College, Datta Meghe Institute of Higher Education and Research, Wardha, IND

**Keywords:** acute motor axonal neuropathy (aman), miller fisher syndrome (mfs), acute sensory and motor axonal polyneuropathy (asman), acute inflammatory demyelinating polyneuropathy (aidp), goal oriented rehabilitation, nprs, functional independence measurement (fim), dass-21 scale, gullian-barre syndrome

## Abstract

Guillain-Barré syndrome is a polyneuropathy that can be caused by an autoimmune condition or a bacterial infection. In typical GBS cases, there is hypo- or areflexia, symmetrical limb weakness that worsens within four weeks of the symptoms. The facial nerve is involved in this situation, which results in weak facial muscles, which, in turn, affect facial emotions and movements. In this case study, a 21-year-old athlete who suffered from unexpected weakness that resulted in quadriplegia had goal-oriented physical therapy treatment designed for the patient, who recovered quickly. This case study aims to emphasize how goal-oriented physical therapy treatment can help patients recover quickly.

## Introduction

The most common cause of acute paralytic neuropathy is known as Guillain-Barré syndrome (GBS), which encompasses a number of clearly identifiable variations [1]. Guillain-Barré syndrome (GBS) is described as an acute peripheral neuropathy that advances over a few days or, at the most, up to four weeks and causes limb paralysis [2]. GBS is an autoimmune condition caused by a previous bacterial or viral infection. Demyelination occurs as a result of immune system responses against target epitopes in Schwann cells or myelin in the acute inflammatory demyelinating polyneuropathy (AIDP) type; however, the precise target molecules in AIDP have not yet been discovered. The prognosis for GBS is typically good, although it is a devastating disease with a death rate of around 10% and a severe disability rate of about 20% [3]. Treatment of GBS is subdivided into (i) the treatment of individuals with severe paralysis who require ventilator support and critical care; and (ii) certain immunomodulating therapies, for example, intravenous infusion of immunoglobulin G, presumably through minimizing nerve damage, to reduce the progressive course of GBS. GBS variations that are often identified include acute inflammatory demyelinating polyneuropathy (AIDP), acute sensory and motor axonal polyneuropathy (ASMAN), Miller Fisher syndrome (MFS), and acute motor axonal neuropathy (AMAN) [4]. GBS has been recorded all around the world and is a prevalent cause of neuromuscular paralysis. There are between 1.65 and 1.79 cases of GBS per 100,000 people per year, according to reports [3]. The majority of research has discovered that the incidence rises linearly with age and that males are approximately 1.5 times more likely than women to be afflicted [5].

## Case presentation

A 21-year-old female who was an active athlete (runner) was admitted to Acharya Vinoba Bhave Rural Hospital (AVBRH) Sawangi Meghe with a sudden episode of weakness in both her arms and legs, which shows that the onset is sudden. She visited a physician, and medications were given to the patient, but there was no improvement in the condition of the patient, so she was referred to Acharya Vinoba Bhave Rural Hospital (AVBRH). The patient was managed with medications, and physiotherapy sessions were regularly given to the patient. For 45 days, the patient was treated at AVBRH.

Clinical findings

The patient had a mesomorphic build and was cooperative. She was well-oriented to time, place, and person. All the vitals of the patient were normal. For both upper and lower limbs, a sensory examination was performed, and there was no sensory involvement. She also had difficulty swallowing, but there were no respiratory complications. Her facial symmetry was also disturbed, which led to the drooling of saliva. On performing sensory examination, superficial, deep, and cortical sensations were intact. All the nerves are intact except the facial nerve. According to the Modified Ashworth Scale, there was no increase in muscle tone in any of the muscle groups. All the reflexes were diminished (Table 1). The muscle strength was assessed using manual muscle testing (Table 2).﻿

**Table 1:** Reflexes examination of the patient.

Reflexes	Right	Left
Biceps jerk	+	+
Triceps jerk	+	+
Supinator jerk	+	+
Knee jerk	+	+
Ankle jerk	+	+
Plantar reflex	Flexor plantar reflex	Flexor plantar reflex

**Table 2:** Manual muscle testing

Muscle Groups	Right	Left
Shoulder Flexor	1/5	1/5
Shoulder Extensor	1/5	1/5
Elbow Flexor	1/5	1/5
Wrist Flexor	1/5	1/5
Wrist Extensor	1/5	1/5
Knee Flexor	1/5	1/5
Knee Extensor	2/5	1/5
Ankle Plantar Flexor	1/5	1/5
Ankle Dorsi Flexor	1/5	1/5

Physiotherapy management

**Figure 1:**
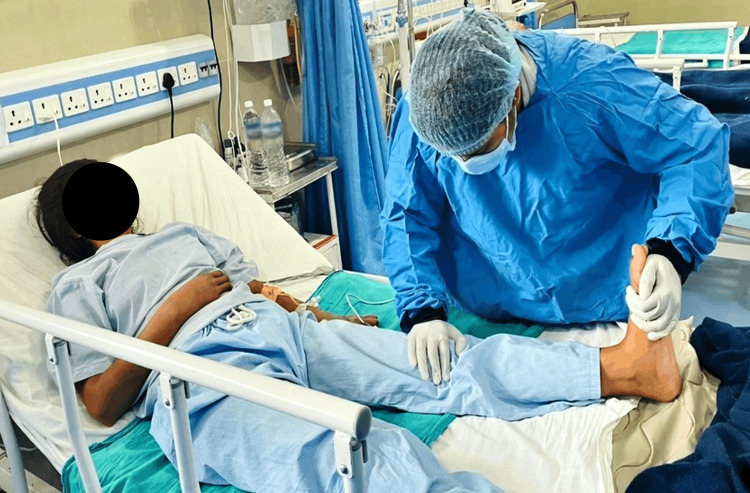
Therapist performing tibialis anterior stretching.

**Figure 2:**
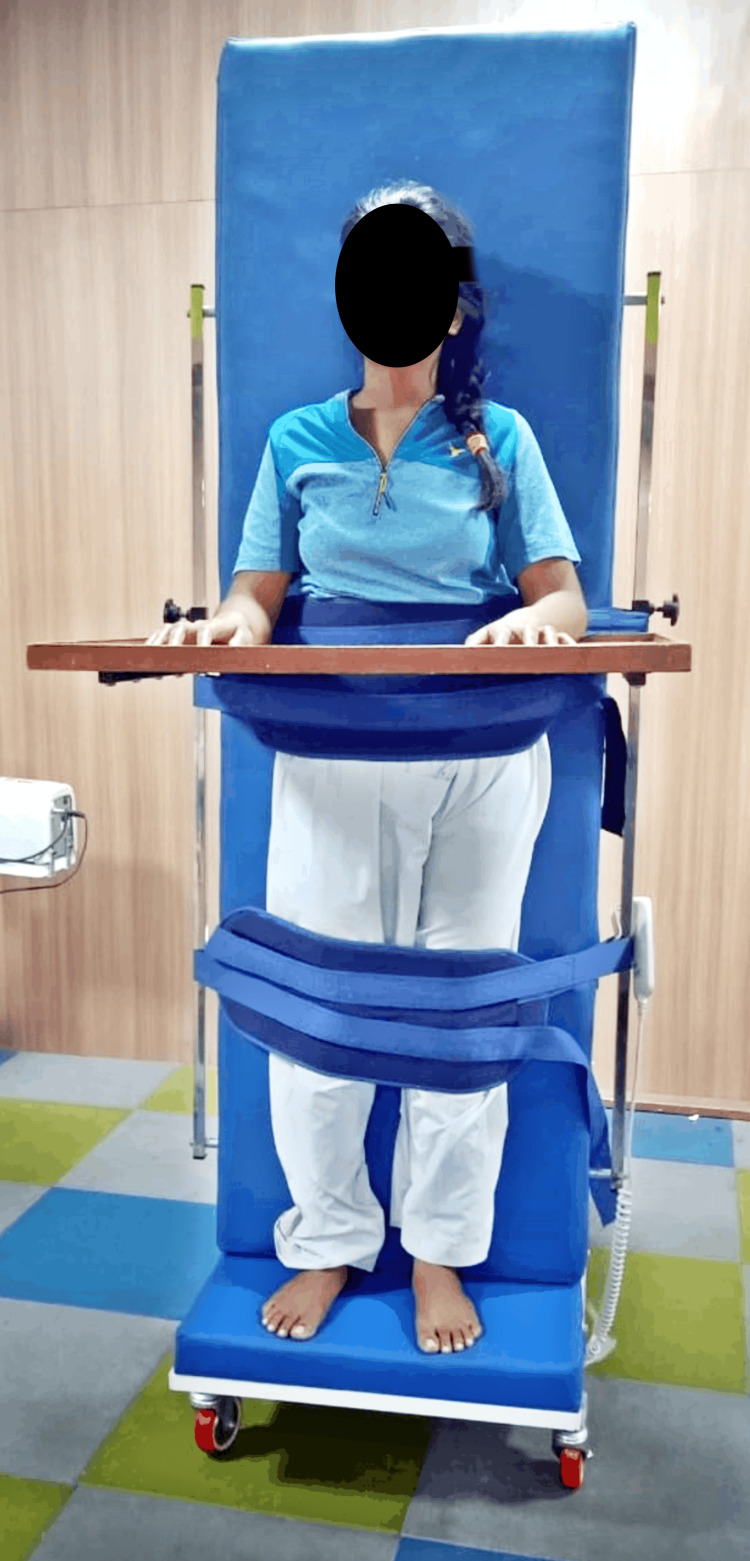
Standing with the help of a tilt table Standing with the help of a tilt table (Shoulder, neutral elbow, 90 degrees extended wrist-neutral knee-neutral ankle-neutral)

Physiotherapy treatment is summarized in Table 3 and Figures 1-2.

**Table 3:** Treatment protocol ROM: range of motion, PNF: proprioceptive neuromuscular facilitation, EMS: electrical muscle stimulator, D: diagonal, min: minute

Intervention	Rationale	Strategy
Patient education	It is important to inform the patient and their family about their condition, the value of rehabilitation, and their exercise routine.	Education about early ambulation, positioning, and giving motivation to the patient.
Passive ROM for both upper and lower limbs	The ROM program must begin during the first few days after hospitalization in order to be successful. It must also incorporate both accessory and physiological movements to boost circulation, lubricate the joints, and preserve the flexibility of the capsular, muscle, and tendon tissues. Two times a day, or more frequently if the patient is not moving at all, passive ROM exercises to the limits of normal range should be done on all joints in the extremities, including the fingers, toes, neck, and trunk.	Passive movements of the shoulder, elbow, and wrist joints. Passive movements of the hip, knee, and ankle joints.
Stretching for hamstrings, adductors, calf muscles	To reduce the tightness of muscles.	Regular sets are given 10 repetitions, and three sets are given.
Pelvic bridging	Help improve motor control and enable pelvic movements.	For 10 repetitions and 1 set.
PNF for upper limb, lower limb and pelvic PNF	To improve neuromuscular effectiveness, flexibility, and range of motion.	D1 and D2 patterns are given for both the upper and lower limbs.
Rolling facilitation	To begin initiating appropriate patient positioning.	Supine to side lying, side lying to prone lying, prone lying to quadripod.
Tilt table exercises	To further take the patient from supine to standing.	For 15 minutes twice a day.
Faradic stimulation	Facial nerve stimulation so that facial movements are initiated by using EMS.	
Facial expression exercises	To improve facial symmetry.	Clinching of teeth, eyebrow exercises, and facial exercises.

Outcome measures

Functional Independence Measure (FIM), NPRS for pain rating, and Depression Anxiety Severity Scale (DASS) were used as outcome measures (Table 4). These are the scores 45 days before and after the treatment. Functional Independence Measure score before treatment was 18, and after treatment, the patient was able to do activities of daily living with moderate assistance. According to the DASS, the depression and anxiety levels of the patient were assessed, and before treatment for depression was at 20, stress was at 25, which is moderate, and after treatment scores for depression were at 13 and anxiety was at 16. ﻿

**Table 4:** NPRS scoring table NPRS: Numerical pain rating scale

NPRS	Score
Pre-treatment	07/10
Post-treatment	04/10

## Discussion

Susanna et al. shared new knowledge about the etiopathogenesis of GBS, which has enabled the development of new treatment strategies that should be started as soon as a diagnosis is made. As summarized in her review of clinical features and the etiopathogenesis of Guillain-Barré syndrome, she claimed that available therapies are insufficient in many patients, particularly in the presence of acute inflammatory demyelinating polyneuropathy [1].

Winer et al. have outlined in their review that recovery from GBS requires a hospital stay and appropriate care. For an early diagnosis and effective treatment of this illness, new developments are even more necessary [4].

Four varieties of GBS and their features have been described by Arthur et al. They also discuss how therapy in the early, acute stages can aid in a successful recovery in all types of GBS [6].

Nehal et al. utilized 20 outcome indicators to describe the function of physical therapy in GBS in a narrative review, and they discovered that GBS can't be treated in a single session; it requires ongoing physiotherapy care. ADLs and functional independence are made possible for patients by effective physical treatment and adequate outcome measurements [7].

Jaee et al. have shown a physiotherapy approach in the early stages of GBS. In their article, it was explained that in atypical GBS, if a proper physiotherapy protocol is given, then full recovery is possible, and hence patients can return to their normal lives [8].

An effective recovery was observed in those getting physiotherapy, according to a national assessment by Ian et al. that contrasted patients with GBS undergoing physical therapy with those who did not [9].

Finisterre listed the advantages of managing physiotherapy to lessen pain and improve physiotherapy regeneration. He also recommended that the length of each session be adjusted in accordance with the degree of symptoms [10].

## Conclusions

GBS is a condition that requires proper and regular physiotherapy management with realistic goals that can be achieved within a particular period. A goal-oriented physiotherapy approach helps to achieve functional independence, due to which the patient can start to go back to his or her normal life and perform ADLs. This goal-oriented approach has been of great importance not only in relieving physical symptoms but also in helping to reduce stress and anxiety in the patient.
